# Proteomic Analysis of Exosomes for Discovery of Protein Biomarkers for Prostate and Bladder Cancer

**DOI:** 10.3390/cancers12092335

**Published:** 2020-08-19

**Authors:** Yi-Ting Wang, Tujin Shi, Sudhir Srivastava, Jacob Kagan, Tao Liu, Karin D. Rodland

**Affiliations:** 1 Biological Sciences Division, Pacific Northwest National Laboratory, Richland, WA 99354, USA; yi-ting.wang@pnnl.gov (Y.-T.W.); tujin.shi@pnnl.gov (T.S.); 2 Cancer Biomarkers Research Group, Division of Cancer Prevention, National Cancer Institute, Bethesda, MD 20892, USA; srivasts@mail.nih.gov (S.S.); kaganj@mail.nih.gov (J.K.); 3 Department of Cell, Developmental, and Cancer Biology, Oregon Health and Science University, Portland, OR 97201, USA

**Keywords:** exosome, proteomics, prostate cancer, bladder cancer, exosome isolation method

## Abstract

Extracellular vesicles (EVs) are released by nearly all cell types as part of normal cell physiology, transporting biological cargo, including nucleic acids and proteins, across the cell membrane. In pathological states such as cancer, EV-derived cargo may mirror the altered state of the cell of origin. Exosomes are the smaller, 50–150 nanometer-sized EVs released from fusion of multivesicular endosomes with the plasma membrane. Exosomes play important roles in cell-cell communication and participate in multiple cancer processes, including invasion and metastasis. Therefore, proteomic analysis of exosomes is a promising approach to discover potential cancer biomarkers, even though it is still at an early stage. Herein, we critically review the advances in exosome isolation methods and their compatibility with mass spectrometry (MS)-based proteomic analysis, as well as studies of exosomes in pathogenesis and progression of prostate and bladder cancer, two common urologic cancers whose incidence rates continue to rise annually. As urological tumors, both urine and blood samples are feasible for noninvasive or minimally invasive analysis. A better understanding of the biological cargo and functions of exosomes via high-throughput proteomics will help provide new insights into complex alterations in cancer and provide potential therapeutic targets and personalized treatment for patients.

## 1. Introduction

Extracellular vesicles (EV) comprise heterogeneous populations of membrane vesicles released by essentially all cell types. They have different sizes ranging from 50–1000 nm in diameter and can be classified into two distinct categories: exosomes and microvesicles. Exosomes (50–150 nm) and microvesicles (50–1000 nm) differ in their modes of biogenesis, but they also share common features, including biological processes (e.g., membrane-trafficking processes and cell adhesion) and biological cargo (e.g., membrane proteins, surface lipids, and nucleic acid) [1] (Table 1). Exosomes are generated within an endosomal system as intraluminal vesicles (ILVs) and secreted during the fusion of multivesicular endosomes (MVEs) with the cell surface [2,3,4], whereas microvesicles are formed by an outward budding at the plasma membrane [5,6]. The nature and abundance of EV cargo [7] are cell-type-specific and involves several physiological states and pathological conditions, including tumorigenesis, inflammation, blood coagulation, and others [1]. In cancer, EVs have been shown to carry tumor-specific proteins and biologically active proteins and to play an important role in premetastatic niche establishment [8,9]. EVs have been increasingly studied recently because they provide a source of relatively low-invasive or non-invasive specimens. EVs can be found in most types of bodily fluids; once EVs are isolated from biological samples (e.g., plasma or urine), proteins or other biomolecules within the EV can be identified and quantified using advanced omics strategies, including both nucleic acid and protein measurements. Although there have been many studies of the RNA cargo within exosomes, examining the protein composition of exosomes provides novel insights regarding both the function and cell site of origin of the exosomes, particularly when post-translational modifications (PTMs) are included in the analysis.

Urologic cancers, including prostate cancer (PCa) and bladder cancer (BCa), are major causes of morbidity and mortality in men [10]. Diagnosis and treatment of these diseases are associated with different but overlapping clinical challenges, and noninvasive or minimally invasive biomarkers are likely to be important for improved clinical practice [11]. This is particularly urgent in the case of screen-detected PCa, where elevated prostate-specific antigen (PSA) levels may be associated with indolent disease which is best treated with active surveillance. An additional application of urinary exosome biomarkers is in the concurrent detection of BCa and PCa, a not uncommon clinical finding [12,13]. Urine represents an excellent source of protein biomarkers for urologic cancers. However, there are large intra- and inter-individual variations in protein concentrations for urine specimens. Urinary EVs have recently been demonstrated to have less variability across different urine samples for identification and quantification of biologically important proteins [14]. Plasma/serum is another ideal source for EVs for minimally invasive identification of cancer biomarkers; however, the presence of high abundance plasma/serum proteins may still affect the analysis of proteins in EVs. Further developments in EV isolation are needed to facilitate clinical applications.

Advanced mass spectrometry (MS)-based proteomics has emerged as a powerful tool for quantitative global proteome profiling of cells, tissues, and biofluids. When integrated with other complementary omics data, proteomic analysis can provide new insights into molecular mechanisms of diseases and identify new biomarkers for diagnosis and/or prognosis and suggest therapeutic targets for better treatment. MS-based proteomic analysis has recently been applied to cancer cell-derived EVs, revealing important biogenesis pathways, and improving our understanding of carcinogenesis and tumor progression [15]. Thousands to tens of thousands of proteins have been reported in various proteomic exosome studies posted to community compendia such as ExoCarta [16] and Vesiclepedia [17,18]. However, it is still challenging to prepare high-purity exosome samples without contamination by other proteins from biofluids or other microvesicles. Because microvesicles are produced by a distinct cellular mechanism and have distinct functions and cargo compared to exosomes, it is essential to separate these two components during proteomic analysis. However, there is currently no analytical method that allows complete separation of exosomes from microvesicles [19] because of the overlapping size, similar morphology, and variable composition [20,21]. Additional challenges for MS-based studies of exosomes include low levels of starting material and varying degrees of dilution. In this review, we will discuss exosome isolation techniques for MS-based proteomic analysis and provide a summary of exosomal protein biomarker studies for PCa and BCa.

## 2. Exosome Isolation and Characterization

**Figure 1:**
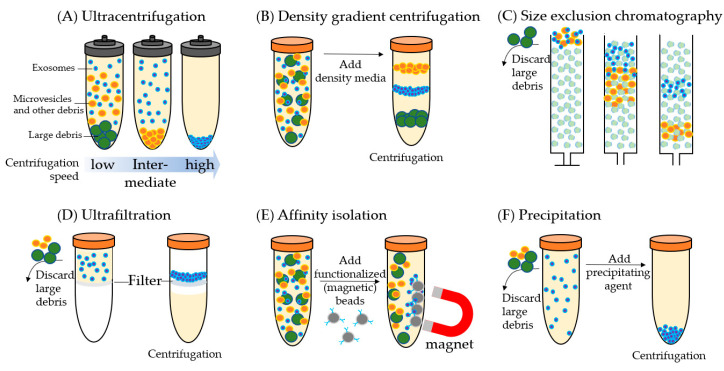
The common methods for isolating exosomes. Blue particles: exosomes; orange particles: microvesicles and other debris; green particles: large debris. (**A**) Ultracentrifugation separation is based on size, and large-size debris and microvesicles are collected earlier at the bottom of the tube and at lower g forces than the smaller exosomes. (**B**) Density gradient centrifugation separation is based on density. Exosomes will travel to their equilibrium density in the centrifugation media. (**C**) Size exclusion chromatography uses a porous matrix (dotted circles) that separates based on size. Soluble components and particles smaller than pore size enter the porous matrix temporarily hence elute later than the particles larger than the pore size. (**D**) In ultrafiltration, soluble proteins and particles smaller than the molecular weight cutoff of the filter (e.g., 105 kDa) are centrifugated through the filter, and the exosomes are collected in the fluid retained by the filter. (**E**) In affinity isolation, exosomes are captured based on their immunophenotype or specific ligands on the surface. Exosomes are often captured using a monoclonal antibody or exosome-specific ligand conjugated to magnetic nanoparticles and captured by magnets. (**F**) In precipitation, the addition of a precipitating agent induces clumping of exosomes and the clumps can be collected by centrifugation. While performing method (**A**,**C**,**D**,**F**) large debris needs to be separated (e.g., by low-speed centrifugation) and discarded before exosome isolation.

Exosomes have distinct biophysical and biochemical characteristics including size, mass density, shape, charge, and antigen exposure which are often used for their isolation from biofluids. There are six common exosome isolation methods (Figure 1): ultracentrifugation (UC), density gradient (DG) centrifugation, size exclusion chromatography (SEC), ultrafiltration, affinity isolation, and precipitation. Each method has advantages and disadvantage but there has been no systematic comparison of all these isolation methods using a single urine or plasma/serum sample. Thus, it is difficult to rank the performance of the different methods (e.g., recovery and purity of isolated exosomes); however, some distinct features of each method are noted.

### 2.1. Ultracentrifugation

The most commonly used method for exosome isolation is UC (Figure 1A). It allows exosomes to be cleared out from intact cells, cell debris, large vesicles, and other contaminants through a series of centrifugation steps. Low-speed centrifugation at 300× *g* is used first to remove large cell fragments and cell debris. Intermediate speed at 2000 g is then aimed at removing cell debris and aggregating biopolymers and the other compositions with density higher than that of EVs. The last step is to pellet extracellular vesicles for the collection of exosomes using high-speed centrifugation at 100,000–200,000× *g* [22,23,24,25].

The UC method has been broadly used for proteomic analysis of exosomes because it can provide high-purity exosomes [26,27,28,29,30,31]. However, it is time-consuming with low sample throughput, and thus it is not practical for analysis of large clinical cohorts. Furthermore, it has a relatively low recovery of EVs due to the sample loss in every centrifugation step [22,25,32,33,34].

### 2.2. Density Gradient Centrifugation

For DG centrifugation (Figure 1B), exosome separation depends on size, mass, and density. A reduced volume of the sample is first loaded on a density gradient medium formed in a centrifuge tube in which density decreases progressively from the bottom to the top, then the DG is subjected to an extended round of UC until density equilibrium is attained. Sucrose and iodixanol are commonly used as DG media to isolate EVs [35,36]. Exosome recovery is ~10–50% depending on the efficiency for removal of the DG medium from the sample [37]. Compared to UC, exosome purity is higher, but the protein yield is lower with DG centrifugation [38,39]. This method is labor-intensive and sometimes requires long-running times to reach equilibrium (i.e., low throughput), and thus is also not suitable for proteomic analysis of large cohorts of clinical samples. Furthermore, since the separation is based on density, the exosome fraction may contain other vesicles of different origins but similar characteristics.

### 2.3. Size Exclusion Chromatography

SEC (Figure 1C) is a popular means for EV enrichment because the majority of EVs are eluted before soluble components (e.g., high-density lipoproteins (HDL)) [40,41,42,43,44,45]. The size cutoff is determined by the choice of the exclusion matrix, such as a pore size of approximately 60 nm for Sepharose 2B. SEC can remove 99% of the soluble plasma/serum proteins and >95% of HDL, resulting in a high-purity fraction of EVs [41]. In addition, mild conditions are used in SEC and thus it does not induce exosome aggregation [45] and retains structural integrity and biological activity of the exosomes [44]. The major co-isolated non-EV components are particles above the size cutoff, which may include viruses, protein aggregates, and low-density LDL [37,41,42,43,44,45]. Using SEC, ~40–90% recovery of exosomes can be achieved with high reproducibility [43], although with low protein yields. SEC enrichment is relatively inexpensive and high throughput (~10–20 min for enrichment and an hour for re-balancing) [42], which makes SEC applicable for large-scale analysis. It has been broadly used in combination with LC-MS for high-throughput proteomic exosome analysis [46,47,48,49].

### 2.4. Ultrafiltration

Ultrafiltration (Figure 1D) allows separation of exosomes from soluble components. Prior to ultrafiltration, samples are filtered through a 0.22-μm filter to remove larger microvesicles and apoptotic bodies [50]. The soluble components are then passed through the filter by applying pressure or by (ultra)centrifugation. Ultrafiltration has a much higher sample throughput when compared to DG centrifugation and UC. It usually takes ~20 min to concentrate over 100 mL of sample [51]; it may also concentrate EVs by up to 240-fold. However, the exosomal protein yield is relatively low, primarily due to the significant loss of highly concentrated exosomes binding to the filters. Practically, ultrafiltration is easily adaptable for high-throughput proteomics analysis with relatively high exosome purity and virtually no limitation of sample volume [50,52,53,54,55,56].

### 2.5. Affinity Isolation

Affinity isolation (Figure 1E) is based on the strong binding affinity of immobilized molecules to specific ligands on the exosome surface. Most immunocapture assays use monoclonal antibodies immobilized on a solid-phase (e.g., magnetic beads) to capture EVs that expose a specific ligand [57,58,59,60]. Other affinity isolation methods use chemical affinity [61,62] or annexin A5 which binds to phosphatidylserine moieties on the surface of most EVs [63]. Based on specific ligands or proteins, immunocapture can isolate subpopulations of EVs [21,64]. Although immunocapture may take several hours for one single enrichment, it can be parallelized in a multi-well plate format for high-throughput analysis [65]. The major limitation of the affinity capture methods is the potential batch effect from the use of magnetic beads and ligand conjugation, which can lead to low reproducibility for proteomic analysis. Another disadvantage is the inability to enrich for more than one subpopulation of EVs at a time. Microfluidic devices have been developed recently for high-throughput affinity enrichment using multi-microchannels and EV arrays [66,67,68,69,70,71,72]. One disadvantage is that microchannels can easily get clogged and it is still time-consuming for each enrichment [73].

### 2.6. Precipitation

Exosome isolation from biofluids can be achieved by altering their solubility or dispersity. This can be achieved using water-excluding polymers (e.g., polyethylene glycol (PEG)) for precipitation of the exosomes (Figure 1F). Polymer-based exosome precipitation is widely used. Large cell fragments and cell debris are first removed by low-speed centrifugation (e.g., 300 g), after which the polymers are added and incubated for a wide range of 15 min to 12 h, depending on the polymers used [74,75]. Exosomes are then enriched by means of either low-speed centrifugation or filtration. This method is easy to set up and is flexible with sample volume. The major disadvantage of polymer-based exosome precipitation is the co-precipitation of abundant non-exosome contaminants, such as proteins and polymeric materials, and hence is not suitable for MS-based proteomics studies [76].

### 2.7. Exosome Characterization for Quality Control

Several techniques are typically used for characterization of the isolated exosomes. By using dynamic light scattering (DLS) and nanoparticle tracking analysis (NTA), the size and distribution of the exosomes can be measured [77]. Transmission electron microscopy (TEM), when combined with immunogold staining, can provide structural detail and delineate the subpopulations of EVs [78]. Cryo-electron microscopy is also suitable for depicting the morphology of EVs because there is no fixation or staining [79]. The combination of scanning electron microscopy (SEM) and atomic force microscopy (AFM) is used to determine the size, morphology, and intactness of EVs [80]. Flow cytometry has been recently demonstrated as a promising tool for qualitative and quantitative analysis of EVs [81]. Western blot, enzyme-linked immunosorbent assay (ELISA), and a photosensitizer-bead detection system (ExoScreen) [82] can also be used to evaluate exosome purity and enrichment efficiency.

## 3. Exosomal Proteins as Biomarkers for PCa and BCa

### 3.1. Overview

PCa is the most commonly diagnosed cancer in men, accounting for 21% of all new cancer diagnoses, and accounts for 10% of all cancer deaths among American men [10]. Although PSA is routinely used for early detection of PCa, the inability of PSA to discriminate between benign prostate diseases and aggressive disease leads to overdiagnosis and unnecessary overtreatment [83,84]. Therefore, there is an urgent need for additional PCa biomarkers for highly specific monitoring of disease progression and treatment response, including noninvasive exosomal biomarkers from biofluids (e.g., urine and plasma/serum). We reviewed recent proteomic studies of exosomes from urine, plasma/serum, and cell culture media for PCa protein biomarker discovery [19,85,86,87,88,89,90,91,92]. A summary of these studies is provided in Table 2. More details are discussed in the following sections.

BCa is the fourth most commonly diagnosed cancer in men, accounting for 7% of all new cancer diagnoses, and has the eighth highest cancer death rate, accounting for 4% of all cancer deaths among American men [10]. Diagnosis of BCa is usually based on cytology, urinalysis, and cystoscopy. Cytology is a highly specific test but with low sensitivity [93]. Cystoscopy is the gold standard for diagnosis of BCa; however, it is invasive, even for flexible cystoscopy, and has the risk of developing urinary infections [94]. Thus, non-invasive and reliable diagnostic biomarkers for BCa are needed. Potential exosomal protein biomarkers from urine and cell lines were recently identified using MS-based proteomics (Table 3). They are promising for both BCa diagnosis and prediction of BCa progression [14,31,95,96,97,98].

### 3.2. Exosome Isolation for Discovery Proteomics

UC, alone or in combination with DG, was the most frequently used procedure for exosomal isolation in the studies reviewed herein. The high degree of purity available in UC and UC + DG preparations is advantageous for MS-based proteomic discovery of exosomal protein biomarkers, but both UC and DG are time-consuming and thus impractical for analysis of large cohorts or direct implementation in the clinic for diagnostic testing [65,99,100] (Table 2 and Table 3). The most comprehensive proteome profiling of urinary exosomes in this review used UC for isolation of exosomes from 18 PCa digital rectal exam (DRE) urine samples [87] (Table 2). By using basic reversed-phase (RP) LC fractionation, 4710 proteins were identified from a total of 58 fractions. The purity of exosomes was confirmed by TEM and the presence of typically enriched exosomal protein biomarkers (e.g., CD9, CD63, and CD81). Gene ontology (GO) cellular component analysis showed that the most abundant proteins that could be derived from EV proteins were plasma membrane proteins (25%). Dhondt et al. used UC + DG for isolation of exosome from 48 urine samples including benign, PCa, BCa, and renal cell carcinoma patients, and TEM and NTA for exosome characterization [85] (Table 2). In total, 3686 proteins were identified with 82% annotated to exosome using GO terms, which demonstrates high-purity exosomes suitable for proteomic analysis. In addition, the average concentration of urinary exosomal proteins was sample-dependent and ranged 0.01–3 µg/mL, so that 10–50 mL of urine was typically required for comprehensive MS-based proteomic analysis.

There are significantly fewer studies on plasma/serum exosomes for PCa or BCa when compared to urinary exosome analysis, presumably because urine is a more relevant biofluid and also less invasive for studying urological cancers. Only two studies were reported for proteomic discovery of PCa exosomal protein biomarkers from patient plasma/serum (Table 2). With ExoQuick or UC, ~300 [92] and ~100 proteins [86] were identified with ~32% and ~70% annotated as exosomal proteins, respectively. The highest number of exosomal proteins in plasma/serum reported so far was 2238 using immunoaffinity isolation with only 5 µL of plasma [61]; however, the GO term analysis showed that only 18.4% were annotated as exosomal proteins, suggesting issues with exosomal purity.

### 3.3. Urinary Exosome Analysis for PCa and BCa

Proteomic analysis of exosomes is still at an early stage and there is no gold standard workflow available in terms of robustness, efficiency, and reproducibility. Furthermore, no consistent trends in the repeated identification of the same biomarkers can be observed among the published studies, most likely due to issues with experimental design including very small sample sets, diverse experimental systems (e.g., urine, plasma, cell lines), and diverse biological endpoints (e.g., presence of PCa or BCa, versus drug resistance in cell lines).

Urine samples collected after DRE may be more enriched for exosomes specifically secreted by prostate cells, and thus contain a higher proportion of prostate-specific proteins [27,101]. Comparative analysis of urine exosomes prior to and after local prostatectomy or radiation by Dhondt et al. revealed a significant reduction in PCa biomarkers proteins (e.g., KLK2, KLK3/PSA, FOLH1, MSMB, ACPP, TGM4, NDRG1, NKX3-1) and androgen-regulated genes (e.g., FKBP5, FAM129A, RAB27A, FASN, NEFH) [102,103] (Table 2). The decrease in abundance of these 13 proteins was consistent with the removal of the prostate gland which acts as a source of these proteins.

Fujita et al. did a comparative proteomic analysis of DRE-urine exosomes from PCa patients at high Gleason score (GS) with negative biopsies as controls, and they discovered 11 differentially expressed PCa protein biomarkers. They were further verified using selected reaction monitoring (SRM)-based targeted proteomics in an independent cohort of 11 negative and 18 PCa positive urine samples [87]. Among the 11 PCa biomarkers, fatty acid-binding protein 5 (FABP5) was confirmed to have higher expression levels in PCa patients and was significantly associated with GS. With further validation by western blot and immunohistochemistry (IHC) analysis, they concluded that urinary exosomal FABP5 could be a potential biomarker that can be used to predict or confirm high-GS PCa prior to prostatectomy.

Øverbye et al. investigated the proteome of urinary exosomes in 16 PCa patients and 15 healthy male controls. 246 proteins were found to be differentially expressed between the two groups, and 37 proteins were prioritized for further analysis [19]. Among the 37 proteins, 17 proteins displayed individual sensitivities above 60% at 100% specificity, and TM256 has the highest sensitivity (94%). Several LAMTOR proteins were also distinctly enriched with high specificity in PCa patient samples. TM256 and LAMTOR1 could be used to augment the sensitivity to 100%. Other promising protein biomarkers were VATL, ADIRF, and several Rab-class members and proteasomal proteins. This study clearly shows the potential of using urinary exosomes to discover protein makers for noninvasive diagnosis and/or prognosis of PCa.

All the above three studies used MS-based proteomics for discovery of urinary exosomal protein biomarkers for PCa, but the overlap of candidate biomarkers across different studies is extremely low, most likely due to differences in study design and endpoints with different sets of urine samples used. Additionally, most of these studies used very small numbers of samples in the discovery experiments (usually 10 or less)—a situation which is conducive to over-fitting and unreliable *p* values. The 13 exosomal protein biomarkers identified as prostate-specific by Dhondt et al. could be identified by Fujita et al., but none of them showed differential expression between PCa patients and healthy controls. Significantly, the two experimental designs were drastically different: Fujita compared patients who were PCa positive to PCa negative patients, while Dhondt et al. compared urine samples before and after prostatectomy, suggesting exosomes in PCa patients may share the same composition as exosomes from normal prostate glands, but PCa may produce more of such exosomes. Similarly, five out of the 11 exosomal biomarkers discovered by Fujita et al. were detected in urinary exosomes analyzed by Dhondt et al. but there was no differentiation power for the other set of urine samples. When these results are compared with another study conducted by Øverbye et al., only three out of the 13 protein biomarkers (FAM129A, KLK3/PSA, and FOLH1) identified by Dhondt et al. and two out of the 11 protein biomarkers (FABP5 and DNPH1) identified by Fujita et al. were identified and they were all upregulated in PCa patients. The LAMTOR family proteins, ADIRF, plastin-2, and Rab-related proteins identified as potential exosomal makers by Øverbye et al. showed no abundance changes between two sets of urine samples used by Fujita et al. (TM256 was not even detected). In addition, different isolation and quantitation methods could also contribute to the biomarker difference. For example, Dhondt et al. used UC + DG, whereas the other two groups used only UC for exosome isolation. Fujita et al. used isobaric tags for relative and absolute quantitation (iTRAQ) based relative quantification, while the other two groups used label-free quantitation method. It is known that the quantitation dynamic range of iTRAQ is smaller than the label-free method [104,105]. Thus, it is possible that many exosomal proteins with small changes were omitted but they may have been sufficiently significant to differentiate cases from controls in the study conducted by Fujita et al.

For BCa urine exosome analysis, Hiltbrunner et al. used paired exosomes from bladder and ureter urine of the same BCa patients to discover BCa-specific exosomal biomarkers (with or without bladder contact) [95]. They discovered several oncoproteins overexpressed in exosomes derived from bladder urine. Forty proteins were significantly overexpressed in bladder urine exosomes, including known oncogenes such as TPP1, TMPRSS2, FOLR1, RALB, and RAB35, while SLC4A1 had lower expression. In addition, in this cohort, three BCa patients had concomitant PCa, but there were no significantly differentiated proteins observed between BCa-only and the concomitant BCa + PCa. Nevertheless, they found that urothelial carcinoma (UCC)-associated proteins (e.g., UPK1A, UPK1B, UPK2, UPK3B) were highly enriched in BCa exosomes when compared to PCa exosomes, but the four proteins showed no specific expression between ureter and bladder urine of BCa or between BCa-only and concomitant BCa + PCa. These studies provide evidence that that selective enrichment of exosomal protein signatures may reflect their corresponding origin of cancer tissue [106,107], rather than cancer versus non-cancer.

Chen et al. performed comparative proteomic analysis of urinary exosomes between nine hernia and nine BCa subjects. 107 proteins showed differential expression between the two sets of samples [96]. Among the 107 proteins, 29 proteins (41 surrogate peptides) were precisely quantified by LC-SRM in an independent small cohort of 48 urine samples (28 BCa, 12 hernia, 5 hematuria, and 3 urinary tract infection/hematuria). 24 proteins were verified to have significantly differential expression between 28 BCa and 12 hernia patients, with individual area under the receiver operator characteristic curve (AUC) values from 0.702 to 0.896. Further analysis of TACSTD2 in the same study using commercially available ELISA in raw urine specimens (*n* = 221) was performed to confirm its potential value for diagnosis of BCa. This study reveals a strong association of TACSTD2 with BCa and highlights its potential clinical utility.

Lee et al. conducted comparative proteomic analysis of urinary exosomes between 10 healthy controls and 10 age-matched BCa patients, and the EV proteome was also compared with the whole urine proteome. MS-based label-free proteomics identified a total of 1222 proteins and 56 proteins were found to have significantly increased expression levels in urinary EVs of BCa patients. Among the 56 protein biomarkers, mucin-1, CEACAM-5, EPS8L2, and moesin were validated with western blot analysis [14]. This study showed that urine EVs have the potential to provide an enriched source of BCa protein biomarkers.

Similar to the above discussion in PCa urine exosome studies, it is difficult to find a common trend for BCa urine exosomal protein biomarkers due to the limited number of reported studies, different objectives, and study design, and different isolation and MS quantitation methods used. The five known oncogene biomarkers (TPP1, TMPRSS2, FOLR1, RALB, and RAB35) discovered by Hiltbrunner et al. did not show differential expression in the other two BCa urinary exosome studies; TMPRSS2 was not even detected in the other two comprehensive proteome profiling studies. Among the 24 exosomal protein biomarkers discovered by Chen et al., TACSTD2 was validated; however, it was not be detected in urine EV analyzed by Lee et al. and showed no abundance changes in exosomes between ureter and bladder urine from the same BCa patients analyzed by Hiltbrunner et al. For the other 23 exosomal protein biomarkers, there were no significant abundance changes for 22 proteins, and one protein was not detected in the study of Hiltbrunner et al. This is not surprising because of the totally different experimental design with different sets of urine samples, as well as the use of small sample numbers which is conducive to over-fitting. More studies and more reliable exosome isolation methods are likely required for the discovery of promising BCa urinary exosomal protein biomarkers which have broad clinical utility. Additionally, attention to standards for quantification and normalization would probably enhance the reproducibility of exosomal proteomics.

### 3.4. Plasma/serum Exosome Analysis for PCa in Ethnically Diverse Populations

Two studies were reported for the discovery of PCa exosomal protein biomarkers in plasma/serum with a focus on different ethnic groups. When compared to other races, African American men face the highest incidence and mortality rates for PCa [108,109]. Thus, it is important to use racially diverse populations for PCa biomarker studies. Using UC for exosome isolation and label-free MS analysis, Panigrahi et al. identified 134 proteins in serum samples from African American men (*n* = 8) and Caucasian men (*n* = 8) with PCa and healthy individuals. Filamin A was found to be a potential biomarker, specifically in African Americans, for discrimination of PCa from healthy individuals, while no Filamin A expression changes were observed for Caucasian men [86]. Turay et al. performed another proteomic analysis of nine healthy controls and 12 PCa patients (four Caucasian, four African American, and four Hispanic) with identification of ~300 proteins [92]. Specific protein biomarkers were identified for PCa patients in each ethnic group when compared to healthy individuals, but Filamin A was not confirmed as an African American specific biomarker, possibly because most proteins identified in plasma/serum exosomes are high abundance proteins in plasma/serum (e.g., albumin, macroglobulin, apolipoproteins, complement component, fibrinogen, haptoglobin, and immunoglobulin G (IgG)), an indication of potential issues in exosome purity. Therefore, prior to plasma/serum exosome isolation, depletion of high abundance proteins may be required, preferably using low-pressure, spin-column type of depletion to avoid exosome damage.

### 3.5. Cell Culture Media Analysis for PCa and BCa

Exosomes isolated from cell culture medium are an interesting model system for discovery of exosomal protein biomarkers and understanding of their biological functions. Unlike urine or plasma/serum where there are many possible tissue sources for exosomes, the exosomes in cell culture media all come from a single cell type. With iTRAQ-based quantitative proteomics *Kawakami* et al. identified and quantified a total of 110 proteins from cell culture medium exosomes in the parental PC-3 cell line and a taxane-resistance PC-3 cell line (PC-3R) [88]. ITGB4 and VCL were found to be upregulated in exosomes derived from PC-3R cells when compared to taxane sensitive PC-3 cells. Biological function studies revealed that silencing of ITGB4 and VCL acted at the level of invasion and migration, not proliferation. Kharaziha et al. identified a total of 914 proteins from culture media exosomes in docetaxel-sensitive and docetaxel-resistant DU145 cells [89]. Unsupervised hierarchical clustering analysis revealed 100 differentially expressed proteins, and MDR-1, MDR-3, Endophilin-A2, and PABP4 were only detected in exosomes from resistant cells. Six patient serum samples were used to validate the four exosomal protein biomarkers. This work demonstrated a useful workflow of using exosomes from PCa cell culture media as an enriched source for biomarker discovery.

Unlike MS-based proteomic analysis, Webber et al. explored a novel aptamer-based SOMAscan array platform for analysis of exosomes from cell culture media in DU145 PCa cell line [90]. Almost 100 proteins from the cell culture media exosomes were found to be significantly differentially expressed relative to the cells themselves (whole cell lysates). Several exosome biomarkers (e.g., NOTCH3, L1CAM, RAC1, and ADAM9) were further validated using western blotting. This result suggests that the SOMAscan array platform can be used as an alternative for quantitative proteomic analysis of exosomal proteins. The protein coverage of the new platform is superior to that of traditional antibody-based immunoassays, but unlike MS-based unbiased analysis, it remains a targeted proteomics approach depending on the availability of aptamer assays for target proteins.

A comparative analysis of the putative PCa exosomal biomarkers identified from cell culture media studies also indicated a lack of consistency. The eight protein biomarkers discovered by Kawakami et al. are in the exosomal protein list from Kharaziha et al. but without quantitative information. Two out of eight protein biomarkers (MSN and RPS27A) were detected in DU145 PCa cell line using the SOMAscan array platform, whereas all the four protein biomarkers discovered by Kharaziha et al. were undetected. This is most likely due to the limitation of the protein array approach with predefined target proteins.

Similar results were observed from comparison of proteomic analyses of exosomes from BCa cell culture media. Silvers et al. discovered one potential BCa biomarker TALDO1 by comparative analysis of exosomes from the muscle-invasive bladder cancer (MIBC), compared to normal urothelial cell cultures [97]. TALDO1 was further verified by IHC staining of BCa tissues. Beckham et al. reported discovery of EDIL-3 as a potential biomarker for high-grade BCa by comparative analysis of exosomes from high-grade BCa with nonmalignant urothelial cell culture media [98]. The function of EDIL-3 for cell migration was also studied by small hairpin RNA (shRNA) gene knockdown and recombinant EDIL-3. Exosomes from cell culture media were also used to study BCa metastasis. Using two isogenic derivate BCa metastatic cell lines Jeppesen et al. identified several proteins linked to epithelial-mesenchymal transition (EMT) (e.g., increased abundance of VIM and HDGF in the membrane exosomes, and CK2 and ANXA2 in the luminal exosomes) [31]. This study highlighted the potential of proteomic analysis of BCa exosomes for better understanding of how proteins from membrane and luminal exosomes are involved in the metastasis process. Attempts to compare exosomal protein biomarkers from the three BCa studies were unsuccessful, possibly due to the use of different BCa cell lines with different treatment conditions.

## 4. Future Perspective

Proteomic analysis of exosomes for protein biomarker discovery is still at an early stage, as evidenced by the relatively few published exosome studies for PCa and BCa. Biomarker development will greatly benefit from more comprehensive and robust characterization of exosomes isolated from much larger cohorts of patient samples; achieving this goal will require improvements in the throughput and reproducibility of exosomal purification protocols, without sacrificing either purity or yield. MS-based proteomics has recently emerged as a powerful tool for both comprehensive proteome profiling and precise protein quantification. Thus, it is widely used for protein biomarker discovery and validation. Using state-of-the-art quantitative proteomics platforms, such as isobaric tandem mass tag (TMT) labeling-based sample multiplexing and basic reversed-phase LC-based fractionation and concatenation [110,111,112], thousands of proteins can be readily identified and quantified, resulting in 10s–100s of candidate protein biomarkers in exosomes from specimens such as biofluid samples. These candidate biomarkers can then be validated by MS-based targeted proteomics platforms. When compared to traditional methods such as western blot and ELISA, MS-based targeted proteomics (e.g., SRM or parallel reaction monitoring, PRM) has significant advantages for biomarker validation in terms of being antibody-independent, high specificity, and high multiplexing (≥200 proteins in a single analysis) [113]. However, for current MS-based proteomic analysis of exosomes, the biggest challenge is still the lack of highly effective and robust methods for rapid, reproducible exosome isolation. UC or combined UC and other isolation methods are typically used to generate high-purity exosomes for discovery proteomics, but these are time-consuming and difficult to maintain high reproducibility for proteomic analysis of large cohorts of clinical samples. Furthermore, they require large volumes of biofluids to have sufficient exosomes for comprehensive proteomic analysis. Future developments will be focused on improvement of rigor and reproducibility for exosome isolation, coupled with improved methods for normalization and quantification, along with improvements in sample throughput through automation and standardization. We anticipate that with significantly improved exosome isolation procedures, advanced MS-based proteomics could facilitate development of exosomal protein biomarkers and their translation into clinical use. Alternatively, the lower-throughput UC methods can be used for the initial discovery of exosomal protein biomarkers in a small cohort, followed by the use of highly reproducible, easily-automated methods (e.g., SEC or affinity isolation) for targeted proteomics validation of the candidate protein biomarkers in large clinical cohorts. Issues with impurity could be effectively addressed by high-specificity targeted proteomics (i.e., MS-based “western blots” [114]) as evidenced by effective LC-SRM analysis of non-depleted plasma [115,116,117].

## 5. Conclusions

Exosomes are released from essentially all cell types and perform diverse cellular functions including intercellular communication, antigen presentation, and transfer of tumorigenic biomolecules. Recently, proteomic analysis of exosomes derived from biofluids has been increasingly used to reveal new protein signatures for noninvasive or minimally invasive cancer diagnosis and prognosis, and to suggest therapeutic targets for better cancer treatment. High-quality and robust exosome enrichment is a prerequisite for effective analysis using MS-based proteomics. In this review, we compared the most common methods used for exosome isolation and discussed their principles, advantages, and limitations. We then summarized recent applications of MS-based proteomics for discovery of exosomal protein biomarkers with a focus on PCa and BCa. Many promising exosomal protein biomarkers have been identified for the two types of cancer either from urine, plasma/serum, or cell culture media. In particular, the potential prostate-specific biomarkers FAM129A, KLK3/PSA, FOLH1, FABP5, and DNPH1 have been observed in three independent publications, albeit with differences in the level of differential expression, and this set is probably the most robust panel identified to date. However, it remains difficult to generalize these biomarkers at present due to issues with small discovery cohort sizes, and differences in analytical platforms and experimental endpoints. Ultimately, these biomarkers will require further verification in large-cohort clinical studies to evaluate their potential clinical utility. Furthermore, more high-quality exosome studies are needed to identify additional promising biomarkers with the potential for broad clinical utility. The major hurdle for the utilization of exosomes and other EVs for cancer diagnosis and prognosis is the lack of robust, reproducible, and high-throughput methods for the isolation of pure EV populations, resulting in low reproducibility in sample quality and potentially misleading results. Automation and standardization of exosome isolation, when combined with advanced MS-based proteomics, may pave the way for rapid, reproducible quantitative proteomic analysis of exosomes, which could bring new momentum to this important research area.

## Figures and Tables

**Table 1:** Comparisons of extracellular vesicle types.

Extracellular Vesicles	Size	Origin	Membrane Protein Biomarkers	Cell-Type Specific Protein Biomarkers	Other Names	Cargo in Common
Exosomes	50–150 nm	Endosome	CD9, CD81, CD63, TSPAN6, TSPAN8, CD151, CD37, CD53, FLOT1/2	HLA, HLA-DQB1, APP, PMEL, TCR, FASLG, CXCR4, HSPG, CD86, PRNP, TFR, WNT	Prostasomes, Tolerosomes, Dexosomes, Nanovesicles, Exosome-like vesicles	Lipids: phosphatidylserine, sphingolipids; Cell adhesion: integrin; Intracellular trafficking: RAB, ANXA; Biogenesis factors: ALIX, TSG101, VPS4; Chaperones: HSP70, HSP90; Nucleic acids: microRNA, other non-coding RNAs, mRNA, DNA
Microvesicles	50–500 nm (up to 1 μm)	Plasma membrane	CD9, CD81, CD82	HLA, LFA1, CD14	Microparticles, Blebbing vesicles, Shedding vesicles, Oncosomes, Apoptotic bodies	

**Table 2:** Proteomic studies for exosome biomarker discovery of prostate cancer.

Potential Biomarker	Cohort	Isolation Method	Proteomics Method	Results	Reference
KLK2, KLK3/PSA, FOLH1, MSMB, ACPP, TGM4, NDRG1, NKX3-1, FKBP5, FAM129A, RAB27A, FASN, NEFH	DRE urine: prior to (*n* = 12) and after local treatment (*n* = 10); prior to RP (*n* = 3); benign prostate hyperplasia (*n* = 12)	Ultracentrifugation and density gradient centrifugation	Label-free	3,686 proteins identified; significant reduction in prostate (cancer)-specific proteins and androgen-regulated proteins after local PCa treatment.	Dhondt et al. [85]
FABP5, Granulin, AMBP, CHMP4A, CHMP4C	DRE urine: discovery cohort (negative, *n* = 6; GS 6 PCa, *n* = 6; GS 8–9 PCa, *n* = 6); validation cohort: negative (*n* = 11); PCa (*n* = 18)	Ultracentrifugation	iTRAQ	4,710 proteins identified; 5 associated with high GS.	Fujita et al. [87]
TM256, LAMTOR1, VATL, ADIRF, Rab proteins, proteasomal proteins	Urine: PCa (*n* = 16); controls (*n* = 15)	Ultracentrifugation	Label-free	1,644 proteins identified; 246 were differentially expressed with a focus list of 37 proteins.	Øverbye et al. [19]
Filamin A	Serum	Ultracentrifugation	Label-free	Filamin A as AA-specific biomarker.	Panigrahi et al. [86]
22 unique for AA; 13 unique for Caucasian; 78 unique for Hispanic	Plasma: PCa (4 Caucasian, 4 AA, 4 Hispanic); healthy controls (*n* = 9)	ExoQuick (precipitation)	Label-free	258 proteins identified; specific protein biomarkers identified for PCa patients in each ethnic group.	Turay et al. [92]
AKAP12, ITGB4, MSN, VCL	Cell culture medium: PC-3 cells and taxane-resistance PC-3 cells	Ultracentrifugation and affinity isolation	iTRAQ	110 proteins detected; ITGB4 and VCL were associated with taxane-resistance in PC-3 cells.	Kawakami et al. [88]
MDR-1, MDR-3, Endophilin-A2, PABP4	Discovery: cell culture median (sensitive and resistant DU145 cells); validation: serum from CRPC patients (3 resistant and 3 sensitive to docetaxel)	Ultracentrifugation and density gradient centrifugation	Label-free	914 proteins identified; 4 proteins were associated with docetaxel resistance in DU145 cells.	Kharaziha et al. [89]
MFG-E8, integrins, MET, ROR1, ITIH4, NOTCH3, L1CAM, RAC1, ADAM9	Culture medium of DU145 PCa cell line compared to whole cell lysate	Density gradient centrifugation	SOMAscan array	~100 proteins enriched in exosomes; NOTCH3, L1CAM, RAC1, and ADAM9 were confirmed by western blot.	Webber et al. [90]

DRE: digital rectal exam; RP: radical prostatectomy; PCa: prostate cancer; GS: Gleason score; iTRAQ: isobaric tags for relative and absolute quantitation; AA: African American; CRPC: castration-resistant prostate cancer.

**Table 3:** Proteomic studies for urine exosome biomarker discovery of bladder cancer.

Potential Biomarker	Cohort	Isolation Method	Proteomics Method	Results	Reference
TPP1, TMPRSS2, FOLR1, RALB, RAB35	9 BCa patients with paired ureter and urine samples (3 also had PCa)	Ultracentrifugation	Label-free	1094 proteins identified; 40 significantly overexpressed in bladder urine; none differentially expressed in BCa patients with concomitant PCa.	Hiltbrunner et al. [95]
TACSTD2	Urine: discovery (BCa patients, *n* = 9; hernia patients, *n* = 9); MRM (BCa, *n* = 28; hernia, *n* = 12; hematuria, *n* = 5; urinary tract infection, *n* = 3); ELISA: BCa with different subgroups (*n* = 140) and hernia (*n* = 81)	Ultracentrifugation	Dimethyl-labeling	2,964 proteins identified; 24 verified by SRM; TACSTD2 further validated by ELISA.	Chen et al. [96]
mucin-1, CEACAM-5, EPS8L2, moesin	Urine: BCa (*n* = 10); healthy controls (*n* = 10)	Ultracentrifugation	Label-free	1,222 proteins identified; 56 proteins significantly increased in BCa; 4 validated by western blot.	Lee et al. [14]
TALDO1	Discovery: cell culture medium (MIBC and normal urothelial cells); western blot: BCa urine (*n* = 6) and healthy control (*n* = 6); IHC: tissue of MIBC (*n* = 51), normal urothelium (*n* = 79), and non-MIBC (*n* = 71)	Ultracentrifugation	Label-free	719 proteins identified; TALDO1 validated by IHC in bladder tissues.	Silvers et al. [97]
EDIL-3	Discovery: cell culture medium from high-grade BCa cell line; western blot: urine from12 BCa patients and 12 healthy controls	Ultracentrifugation	Label-free	453 proteins identified; EDIL-3 validated in BCa patient urine by western blot.	Beckham et al. [98]
VIM, HDGF, CK2, ANXA2	Cell culture medium from BCa cell line and its two isogenic metastatic and nonmetastatic cell lines	Ultracentrifugation	iTRAQ	>1000 and >500 were identified in the membrane and lumen fractions of exosome, respectively. 4 linked to EMT in metastatic cells.	Jeppesen et al. [31]

BCa, bladder cancer; PCa: prostate cancer; SRM: selected reaction monitoring; ELISA: enzyme-linked immunosorbent assay; MIBC: muscle-invasive bladder cancer; IHC: immunohistochemistry; iTRAQ: isobaric tags for relative and absolute quantitation; EMT: epithelial-mesenchymal transition.
